# Identity and research ethos in Indigenous-to-Indigenous planning research

**DOI:** 10.3389/frma.2023.1118038

**Published:** 2023-02-09

**Authors:** Michelle Thompson-Fawcett

**Affiliations:** School of Geography, University of Otago, Dunedin, New Zealand

**Keywords:** Indigenous planning, Indigenous research, research ethics, research sovereignty, Indigenous identity, research across Indigenous boundaries

## Abstract

As a member of an Indigenous community myself, my research is necessarily undertaken through an emancipatory Indigenist methodological approach. Indigenous methodologies seek to deconstruct Western paradigms of investigation and understandings that perpetuate the invalidation of Indigeneity, and instead attempt to constitute paradigms centred on Indigenous worldviews. However, Indigenous researchers often work with communities that are not their own. In my case, I have collaborated in a small amount of research with Indigenous groups outside of my own country. But, the majority of my research has been with New Zealand Māori communities other than my own. Key for me, has been the development of personal strategies aimed at keeping me culturally safe in my research with other Indigenous communities, while being secure in my own Indigenous identity. I seek to be culturally respectful in the space of others - safeguarding local Indigenous research sovereignty.

As Māori, when we introduce ourselves we begin with a short Indigenous expression of our identity. This tribal saying allows those who do not know us in New Zealand's Indigenous communities to easily understand where to place us locationally and ancestrally. This statement is a 3-dimensional indication of our genealogical connection—the words signal our identity through an explanation of our genealogy and link to the place/s with which we have enduring ancestral connection. So, we begin by acknowledging our ancestors and ancestral environment—our integration with lands, waters, species, and the spiritual and metaphysical. We acknowledge the places that embrace and nurture our people. These treasures are testament to the longevity of our people, and the memory of tribal elders who have left a legacy for the living to uphold ancestry and customs unique to our ancestral lands, waters and people.

By reciting genealogy through the history and places in which our ancestors lived and were nurtured, we conceptualize where we belong and our integration with the physical and spiritual realms. This belonging is woven through our life *via* narratives, physical spaces, common practices, and shared values. It is a genealogical connection between where we have a place to stand ancestrally, where our ancestors once stood, and where our future generations will stand, that forms a sense of place and identity across the many strands of the family and tribe to which we belong. Maori futures are contextualized by our genealogy. Honoring and respecting this is essential as part of our research practice and guarding of research sovereignty.

## Ngāti Whātua Orākei

In developing the research activities with which I am involved, a pivotal moment for me occurred 40 years ago in my own neighborhood: an aggressive and racist expulsion by police and army of a peaceful 18-month land occupation protesting against plans for an up-market housing development on the last 25 hectares of Crown land that one of the Ngāti Whātua sub-tribes had hoped would be returned to it. 222 protestors were arrested for “trespassing” on unceded ancestral lands, and the temporary meeting house, buildings and gardens demolished. The completely fraught, enduring, haunting history of the land around this Ōrākei/Okahu Bay area tells a damning story about how the city of Auckland was created, and has been reproduced ever since.

I was a school child living less than 2 km away from all this activity as it took place. I was cognisant of the tormenting history from tangata whenua being deliberately displaced from their turangawaewae (standing place) and identity in Ōrākei, as part of a century-long colonial praxis of dispossession and displacement. As I watched all this unfold, it seemed to me that the ongoing politics of place, and power injustices linked to the control of space, were among the most important issues you could seek to unveil in our society.

## My work and research agenda

Hence, my endeavors in the academy (in the disciplines of geography and planning) and with Indigenous communities prioritize:

- uncovering the importance of place in the maintenance of (cultural) identity;
- revealing the power relations evident in the practices surrounding space;
- then envisioning transformation that will facilitate the aspirations of decolonisation.

This work is not objective or neutral—I do not sit outside of the research experience. The research purposefully challenges behavior, culture, structure and governance, with a view to being emancipatory and transformative through the espousing of Indigenous worldviews, solutions, methodologies, ontologies, epistemologies and axiologies.

As an Indigenous researcher, I am steadfastly committed to working at a grounded level with tribal and sub-tribal communities—whether my own or others with whom I have ongoing associations. This inevitably means my research is deeply personal, and involves emotional and spiritual connection with Indigenous people, communities, and their histories and practices. But because I work within the Western establishment of the University, I also have to work hard to maintain credibility with my Indigenous roots.

However, given that I have lived at least 1,500 km from my tribe for most of the last 35 years, in fact the majority of my research is based outside of my tribal area, and with other tribal groups with whom I have longstanding relationships, who approach me and my Indigenous collaborators to research with and for them in regard to matters of Indigenous planning and development … their aspirations and priorities for planning. This results in a complex Indigenous-Indigenous union, where I am the outsider, the guest; where I am working across Indigenous boundaries and experiences (Smith, 2014). I must constantly reflect on who I am (maintaining my own Indigenous identity) and how I am (humbly) playing the role I have in the research, and how the research sovereignty of the local Indigenous community is maintained. This can be quite tricky, primarily due to the significant and persistent pressure from non-Indigenous researchers for me to assist their teams incorporate Indigenous elements into their research programmes, commonly with limited understanding of an Indigenous-centered research ethos.

While there is a growing and vibrant literature addressing Indigenous approaches to research (e.g., Drawson et al., 2017; Chilisa, 2019; George et al., 2020; Ryder et al., 2020; Smith, 2021), I can find very little mention of the nuances of engaging in Indigenous-to-Indigenous research activity. So, in the following sections, I hope to provide insight into the connection between my identity, my research ethos, and the complexities of Indigenous-Indigenous research, based on a series of research projects with tribes other than my own.

## Introduction to the research activity

By way of a brief background for this paper, here I note the planning-identity-wellbeing nexus of my recent research from which the ideas in this paper are drawn. Holistic Indigenous understandings of wellbeing typically integrate health and development with the physical, spiritual and community environment where a people have their ancestral standing. Accordingly, Indigenous people's meaning in life and self-worth have a particular connection to the place of their historic belonging. Their actions and behaviors may reflect an ancestral integration with landforms and may be manifest in physical and spiritual practices. However, most research on Indigenous wellbeing has been founded on non-Indigenous notions of health rather than broader Indigenous conceptions of wellbeing. In recent research, I have been exploring the associations between the wellbeing of Indigenous communities and their identity in place in the settler-colonial urban design context.

In that context, I suggest that recovering and refreshing a traditional ethic of “locatedness” would be highly beneficial in (and for) twenty-first century planning. Such an ethic would embrace the distinctiveness of land, language, histories and culture while reawakening a focus on the holistic wellbeing of Indigenous communities commensurate with (post)-colonial planning needs. Although dominant colonial practices reproduce Western ways of being in urban planning and design, in recent decades there has been a significant resurgence of resistance through the application of Indigenous knowledges and praxes.

My research on Indigenous initiatives demonstrates there is much to be gained by empowered Indigenous communities who facilitate and deliver wellbeing and design that is contextualized to place and identity, “in accordance with their own cultural aspirations—geographically positioned, historically embedded, holistically interconnected, consciously specific, but also continually negotiated” (Thompson-Fawcett and Quigg, 2017, p. 231). Such Indigenous-led transformation also has repercussions for wider settler-colonial society, which needs to be informed, and reformed, by the wero (challenge) placed in front of it by the Indigenous world (ibid.). The basis for optimism comes from the success of initiatives that reassert the potency and integrity of Indigenous philosophies and actions. These philosophies and actions challenge how broader society can envisage the wellbeing of Indigenous peoples in colonized locations as part of the urban design, development and planning process. They also challenge dominant society's recognition of treaty partnership, Indigenous sovereignty, and Indigenous self-determination.

But, the focus of this paper is not the substance of that research; it is the ways of being during the research process.

## My Indigenist methodological approach

As a member of an Indigenous community myself, my research is necessarily undertaken through an emancipatory Indigenist methodological approach. Indigenous methodologies seek to deconstruct Western paradigms of investigation and understandings that perpetuate the invalidation of Indigeneity, and instead attempt to constitute paradigms centered on Indigenous worldviews (e.g., Romero-Little, 2006; Kovach, 2021).

Indigenous methodologies and research practices are as varied as the diversity of Indigenous communities; developed as appropriate to the particular ways of being and knowing in each Indigenous context. Such indigenising of research recognizes the situation of colonized Indigenous communities in terms of their recovery of practices, rights and histories; ambitions for development; and endeavors for self-determination. It also recognizes the need to make space for Indigenous sovereignty over research related to Indigenous peoples.

Any related research agenda necessarily involves processes of decolonisation, healing, mobilization and transformation—reclaiming power and identity (Smith, 2021). While Indigenist approaches are not prescriptive, they tend to be firm on the research being Indigenous centered and collectively designed, defined, controlled and owned (in the sense of guarded or cared for) by Indigenous communities, thus retaining a strong contextual orientation.

This means that the research questions asked are those chosen and prioritized by the Indigenous community, rather than those given priority by external researchers or funders. For example, the Kaupapa Māori research approach my research is built on is grounded in Indigenous knowledge, principles, language, culture and wellbeing (Smith, 1990). The research practices are founded in respect, being present, watching, listening, and acting with generosity, circumspection, dignity, acknowledgment, humility and giving appropriate attribution (Smith, 2021).

Furthermore, the research is embedded in lasting relationships with, accountability to, and outcomes for the Indigenous community with whom the research is being undertaken (Smith, 2021). In this way, Kaupapa Māori research has been crucial in facilitating a new trust in research – trust that was previously absent due to self-serving Western practices of research on Māori.

Many aspects of the Kaupapa Māori approach resonate with approaches taken in other Indigenous communities, such as Indigenist Australian research frameworks (Rigney, 1999), and the “R's” of First Nations research methodologies—respect, relevance, reciprocity, responsibility (Kirkness and Barnhardt, 1991). This is important because Indigenous researchers often work in communities that are not their own. In my own case, I have collaborated in a small amount of research with First Nations in British Columbia and Manitoba. However, the majority of my research has been with Māori communities other than my own.

## A manawhenua Development Trust

Early in my career I was a local authority planner in South Auckland. As a division of the organization, the Planning unit had close ties with certain local sub-tribes. When staff from a local manawhenua Development Trust (an environmental organization within one of the local tribes), with whom we had meaningful connections, interacted with me as the sole Māori planner in the local authority, they suggested to the divisional leader that they wished they had a clone of me on their own staff to develop an Indigenous management plan. Shortly afterwards, an arrangement was made for me to facilitate the preparation of that plan at the Trust's offices, with some additional Council funded staffing—a kind of secondment. In working with elders on the purpose and directions of that document, I remember my silent gasp when one of the aunties said during a preparatory meeting “It's good that we have you here with us dear, but it would be much better if we had one of our own doing this job instead”. In part, that may have been simply because I was not family; it may have been that elders would feel safer revealing—or freer to reveal—their aspirations to family; it may have been because I did not know the practices and stories of this community; it may have been that she wished one of their own was learning from the elders in the way I was privileged to through this process. Or all of the above. In the end, after more than a year of meetings and honing, a concise Indigenous management plan was produced. It was wider in scope than merely an environmental plan—covering justice, health, social, cultural and economic ambitions as well. And as soon as it had been produced, the Trust took back full control and guardianship of the plan by retaining it as an internal document, not for sharing with my employer or other local authorities in their tribal territory. It was a difficult conversation that I had with my Council supervisor following this reclaiming of the document by the Trust. We (the local authority) had not anticipated that outcome. But it certainly made me ponder those questions: who am I, what is my identity, what role am I playing in an Indigenous-Indigenous situation and how; and what might genuine Indigenous research sovereignty entail?

## Researcher choices: An apprehensive being

Key for me, then, has been the development of personal strategies aimed at keeping me culturally safe in my research with tribes other than my own, while being secure (although often necessarily subtle) in my own Māori identity, and culturally respectful in the space of others. I take a very cautious, perhaps overly-cautious approach—especially when compared to younger Māori researchers raised in the post-1980s Māori language/schooling revival era. My strategies for undertaking Indigenous-to-Indigenous research include:

- Only taking on projects initiated by tribal/sub-tribal communities (not initiating projects based on my priorities but the local Indigenous community's priorities, i.e., start with relinquishing researcher power as per Smith, 2021)
- Building respectful relationships slowly and establishing a dialogue about how to proceed to achieve mutual learning, information sharing and collaborative knowledge creation on the Indigenous community's terms (Ball and Janyst, 2008; George et al., 2020)
- Maintaining long-term relationships with those communities, not just for a defined research period, and carefully protecting those relationships (e.g., Sherwood et al., 2015)
- Establishing an advisory group of community and academic mentors with experience in researching by and with Māori communities
- Taking a team approach on the academic side as well, so that I am not the only Indigenous academic researcher involved
- Ensuring there are academic researchers on the project from the particular tribe/sub-tribe with whom we are working
- Avoiding trying to rush—working slowly, holding back my own Indigenous assumptions, recognizing I do not know the local Indigenous histories, experiences, practices (Smith, 2014)
- Publishing slowly—disseminating research outcomes appropriately and meaningfully, with the Indigenous community first
- Working with Indigenous students to foster their planning-related development with their own tribe/sub-tribe (with whom we are working)
- Deflecting non-Indigenous students from devising their own projects to work directly with Indigenous communities
- Applying Kaupapa Māori Research principles, practices and ethics (e.g., Smith, 2000, 2013; Pihama et al., 2002)

I know others in my position will have their own strategies, some similar, some rather different. A commonality, I expect, will be the priority given to understanding matters of Indigenous research sovereignty related to Indigenous self-determination. I now give a couple of examples of how employing (or not) the above strategies has delivered in practice. First, an example that worked well, and second an example that perhaps demonstrates I should have kept more firmly to my strategies instead of being persuaded otherwise by my non-Indigenous colleagues.

### Māori and mining

In 2011, some of the southern sub-tribal groups in Aotearoa New Zealand were being courted by major international mining companies who wanted to engage in off-shore exploration and possibly extraction along the tribal shoreline and waters. The companies arranged meetings in tribal meeting spaces and other spaces to elaborate on their plans, undertake consultation, and receive feedback. One of these meetings was at one of the local traditional meeting houses in the wider Dunedin area. Several academic staff from my university belong to that Indigenous community and attended the tribal meetings. This was a community several others of us had worked with in a variety of capacities over many years. In talking afterwards, some tribe members expressed the desire for expanded knowledge of mining processes and their impacts in order to engage meaningfully on the issues at hand. In conversing with academic tribal members it was agreed it would be useful to have researchers look into the processes, impacts and possible ways forward. From that was borne a multi-disciplinary group of researchers, primarily Māori, but mostly not from the local tribe, tasked with producing materials that would assist the sub-tribe in their deliberations. One of the tasks was also to see what other tribes around the country were doing in regard to extractive industry. These tribes were all in different positions, but most expressed a strong desire to also be the recipients of whatever information and guidance our team produced. After more than a year of research, the team, consisting of university policy analysts, scientific illustrators, graduate researchers, and professors in law, planning, geology, Indigenous studies, and environmental studies, produced an easy read, well illustrated guidebook. The guidebook covered mining processes, application of relevant Indigenous values by other tribes, analysis of mineral law, evaluation of economic implications, and discussion of environmental impacts from mining. There was a formal booklet launch and local tribes and tribes around the country, along with government departments, local authorities, environmental agencies and petrochemical companies eagerly snapped-up the guidebook. Following the launch, the research team broadened their connections to other universities, met again several times with elders both on and off the tribal land, and prepared an academic presentation for an international Indigenous conference and published other resulting papers. In addition, the initiating sub-tribal group has subsequently developed a detailed in-house policy document on mining in conjunction with tribal members and tribal academics from our team; and to satisfy my employer, I was also able to publish a scholarly piece on the concept of Social License to Operate with an academic from this local tribe and a Māori graduate student.

From my perspective, this was a project that worked well and that enabled my contribution in a culturally safe environment. It was the embodiment of my above research strategy, and a good example of Kaupapa Māori research: research prioritized and defined by an Indigenous community; that valued Indigenous knowledge, principles, practice and wellbeing; and that was based on longstanding relationships and accountability to the local sub-tribe; and that safeguarded local Indigenous research sovereignty. But this is not always how things work out for me.

### Rural development

In early 2017 I was approached by three non-Indigenous colleagues to join them in a new research project about understanding the potential of rural New Zealand. They were quite a long way down the grant application path. While I had done a tiny amount of unpublished research in this area, I was not clear why they were inviting me to join them at this late stage in their preparations. I considered their proposal, but decided it did not sit within my core research interests, and given how many projects I was already involved with, I did not need the extra load (there is no shortage of demand for Māori researchers due to most externally funded grants in New Zealand being required to consider the relevance of the work to Māori).

Several months later, that research team were awarded the grant with the proviso that they include a Māori researcher and adapt the nature of their project accordingly. Now I understood why they had approached me earlier in the year, although the reasoning was not disclosed at the time. The funders approached me directly and strongly encouraged me to join the team. Reluctantly I agreed, but never felt meaningfully welcomed as a real member of the team—perhaps largely because I seemed incapable of conceiving how they might operate respectfully and appropriately with the Indigenous communities, given that those communities had not played a foundational part in establishing the research agenda.

The team had a pacy approach to undertaking research (which is difficult to weave into an Indigenous context), and hoped I could help them establish connections with the local Indigenous communities in order to set up meetings to discuss rural futures. I found this very awkward. Why would the busy sub-tribe members see any point in handing over their visions to this researcher team? I had to work hard to stop haste and a Western agenda from potentially damaging university relationships with the local Indigenous communities.

One key in researching with Indigenous groups in my experience, is bringing those groups into the initial conceptualizing and designing of the research from the outset. I had tried to encourage these researchers to understand what that meant, but I just did not seem to be able to offer them clarity on the rationale and what it meant in practice. So, in this case, the researchers were trying to fit the Indigenous groups into their existing agenda. I tried to think of a way around this. Then I recalled a member of one of the local sub-tribes saying he was interested in further postgraduate study into the development of tribal housing located within the main tribal meeting hub, as this had been an aspiration the local tribe had had for some time. So I suggested to the research team, that this could perhaps be a more appropriate root in for their research; a root that had been identified and prioritized already and in regard to which I had some skills to offer.

They were keen to get moving and wanted to set up meetings immediately. I asked them to set up a meeting first with the sub-tribal environment agency so we could see where things were up to with tribal housing instead of rushing straight into conversations with tribal members. We had that meeting and the team was again keen to push forward with progress. Yet I still had some questions for them. What exactly were they planning to offer these communities? How would we reciprocate and deliver something in return in regard to the housing ambitions? It became clear that that part of the task was going to sit on my shoulders and I was not convinced that if we went in and asked how we could help with achieving this dream that any of my colleagues were going to be with me to work on this in the future. It seemed like far too big a project for me on my own, given my existing projects and role as Head of School. So, I pulled right back. I had come on board to assist with their research, but not to run a programme of my own. It appeared to me that their priority was to focus on what they had applied for in the research grant, and the Māori add-on would be my sole responsibility once preliminary conversations had been started. This was beyond what I felt I had agreed to in connecting with the project to assist its broadening of agenda.

In the end, the only contribution I made was in working with one of my Master's students on a small-scale project examining Indigenous development activity in a remote community. It delivered a thesis and a joint journal article by the student and me—and met the basic requirements in terms of outputs for the grant.

Overall, I found the experience wounding and also demoralizing, especially because I do not believe that I made any headway in terms of encouraging my colleagues to embrace research engagement with Indigenous communities. I feel I was lucky to get away without harming my relationships with the local Indigenous communities by promising more than could be delivered. In the end, I stayed true to my identity, clawed back my usual research strategy with Indigenous communities, and frustrated my colleagues. In the process, there was no breach of Indigenous research sovereignty.

## Being (and the wellbeing of) the research outsider

As you can see from my approach and the two latter research stories, I have chosen to tred a very careful, tentative path in my research activities. I have always suspected that I am too reluctant as a researcher. Most researchers I know are much bolder. I wonder if I over-compensate to avoid appearing to be a colonized “Indigenous” researcher, based in the Western Academy, operating within a non-Indigenous paradigm, and effectively mirroring a privileged white scholar. So, I try as far as possible, to assume the agenda of the Indigenous community rather than the agenda of the academy.

This is a path through which I achieve safety and by which my wellbeing and sense of identity is maintained—based on my ancestral belonging being far away from where I actually conduct research. My acute awareness of who I am when I am researching with communities helps me watch, listen, reflect and remain humble as I walk with Indigenous groups to which I do not belong.

Nevertheless, It certainly has not stopped me from making a big noise at the other end of the research process in terms of seeking transformative planning practice and Indigenous self-determination by:

- contributing to the endeavors prioritized by Indigenous communities;.
- calling to account local and central governments and agencies;
- enabling Indigenous planning students to be Indigenous-planning-knowledge learners; and
- encouraging non-Indigenous students to appreciate Indigenous ways of knowing and what that means for our natural and physical environment.

In this way, I can remain true to my identity: my strength is not as an individual but as part of a collective. And that collective is not always my home collective; its dominion requires appropriate respect.

## Data availability statement

The original contributions presented in the study are included in the article/supplementary material, further inquiries can be directed to the corresponding author.

## Ethics statement

Ethical approval was not required for the study involving human data in accordance with the local legislation and institutional requirements. Written informed consent to participate in this study was not required in accordance with the national legislation and the institutional requirements.

## Author contributions

The author confirms being the sole contributor of this work and has approved it for publication.
